# Membrane contacts with the endoplasmic reticulum modulate plastid morphology and behaviour

**DOI:** 10.3389/fpls.2023.1293906

**Published:** 2023-12-04

**Authors:** Jaideep Mathur, Thomas Kadanthottu Kunjumon, Alena Mammone, Neeta Mathur

**Affiliations:** Laboratory of Plant Development and Interactions, Department of Molecular and Cellular Biology, University of Guelph, Guelph, ON, Canada

**Keywords:** membrane-contact-sites, chloroplasts, endoplasmic reticulum, stromules, lipases, BnCLIP1

## Abstract

Plastid behaviour often occurs in tandem with endoplasmic reticulum (ER) dynamics. In order to understand the underlying basis for such linked behaviour we have used time-lapse imaging-based analysis of plastid movement and pleomorphy, including the extension and retraction of stromules. Stable transgenic plants that simultaneously express fluorescent fusion proteins targeted to the plastid stroma, and the ER along with BnCLIP1-eGFP, an independent plastid envelope localized membrane contact site (MCS) marker were utilized. Our experiments strongly suggest that transient MCS formed between the plastid envelope and the ER are responsible for their concomitant behaviour.

## Introduction

Plastids, with postulated endo-symbiogenic origins are a major defining organelle of the viridiplantae, and best studied in the form of chloroplasts (Archibald, 2009; Keeling, 2010). Like other organelles, plastids move around the plant cell as part of the general actin-myosin dependent cytoplasmic stream (Ueda et al., 2010; Duan and Tominaga, 2018). However, an additional behavioural feature associated with single plastids in green plants is the sporadic extension of their double membrane envelope into thin tubules (Wildman et al., 1962; Gunning, 2005; Mathur et al., 2022). Observations made using a stroma-targeted Green Fluorescent Protein (GFP) recognized the dynamic extensions as stroma-filled tubules and named them as stromules (Köhler et al., 1997; Köhler and Hanson, 2000). Stromules have been shown to extend along cytoskeletal elements (Kwok and Hanson, 2003; Erickson and Schattat, 2018; Erickson et al., 2018; Kumar et al., 2018) but specific proteins linking them to the cytoskeleton have yet to be identified. Stromules have also been shown to align with the endoplasmic-reticulum (ER) membranes and often form branching patterns reminiscent of the ER mesh (Schattat et al., 2011a; Schattat et al., 2011b). Tandem behaviour of stromules and the ER provided a strong basis for speculations about the existence of membrane contacts sites (MCS) between the plastid envelope and neighbouring ER (Wang and Benning, 2012; Pérez-Sancho et al., 2015; Mathur et al., 2022).

The evidence for plastid-ER MCS has come from several transmission electron microscopy-based investigations that point to regions of apposition between the plastid outer envelope membrane (OEM) and the neighbouring ER (Wooding and Northcote, 1965a; Wooding and Northcote, 1965b; Diers, 1966; Crotty and Ledbetter, 1973; Whatley, 1977; Whatley et al., 1991; Kaneko and Keegstra, 1996). By using targeted fluorescent proteins a loose, dynamic ER cage has been observed around each plastid (Griffing, 2011; Schattat et al., 2011a; Schattat et al., 2011b; Mathur, 2021; Mathur et al., 2022) while laser-assisted optical manipulation has established that considerable pulling force of up to 400 pN is required for separating chloroplasts from the ER (Andersson et al., 2007). From a functional viewpoint plastid-ER MCS are strongly implicated in the bi-directional trafficking and exchange of acyl-lipids (Wang and Benning, 2012; Michaud and Jouhet, 2019).

Investigations on other organelles in non-plant systems have shown that MCS formation may have specific consequences for shape modulation and positioning for at least one of the interacting organelles (Prinz, 2014; Prinz et al., 2020). Thus, the motor-driven movement of one organelle may affect the positioning of another organelle connected to it at the MCS (Knoblach et al., 2013; Valm et al., 2017; Guo et al., 2018). Whether a similar MCS-mediated situation exists for the positioning and pleomorphy of plastids has remained un-investigated so far.

An important tool for investigating plastid-ER relationship including the formation of MCS has become available in the form of a putative *Brassica napus* chloroplast-localized lipase fused to eGFP (BnCLIP1-eGFP; Tan et al., 2011). Despite missing unequivocal support for the purported lipase activity of BnCLIP1, several publications have presented it as a general TAG lipase and speculated about its functions (Block and Jouhet, 2015; Mueller-Schuessele and Michaud, 2018; Michaud and Jouhet, 2019; Li et al., 2020; Leterme and Michaud, 2022).Transient expression of BnCLIP1-eGFP in tobacco leaf tissue revealed a clear punctate localization on the plastid envelope. Observations of ER membranes in proximity to the BnCLIP1-eGFP punctae strongly suggested it as a potential candidate protein for MCS formation (Tan et al., 2011). However, the transient nature of experiments reported by Tan et al. (2011) using this probe precluded long-term assessment of its utility for establishing the consequences of plastid-ER membrane interactivity.

Here, based on its demonstrated interactivity with the ER (Tan et al., 2011) and its citation as a MCS marker (Block and Jouhet, 2015; Michaud and Jouhet, 2019; Leterme and Michaud, 2022), we generated stable transgenic lines of *Arabidopsis thaliana* expressing BnCLIP1-eGFP. As shown here, we have used this important fiducial marker for investigating plastid-ER interactions. Our investigations establish that stochastically localized BnCLIP1-eGFP enriched punctae on the plastid envelope form small regions where plastid and ER membranes become linked. The dynamic behaviour resulting from such linkage affects plastid movement and relocation as well as the extension and retraction of plastid tubules called stromules. Our observations provide strong cell biological evidence that underscores the role of MCS with ER in dictating plastid behaviour in plants.

## Results

### BnCLIP1-eGFP patches localize on the chloroplast envelope in transgenic Arabidopsis plants

**Figure 1:**
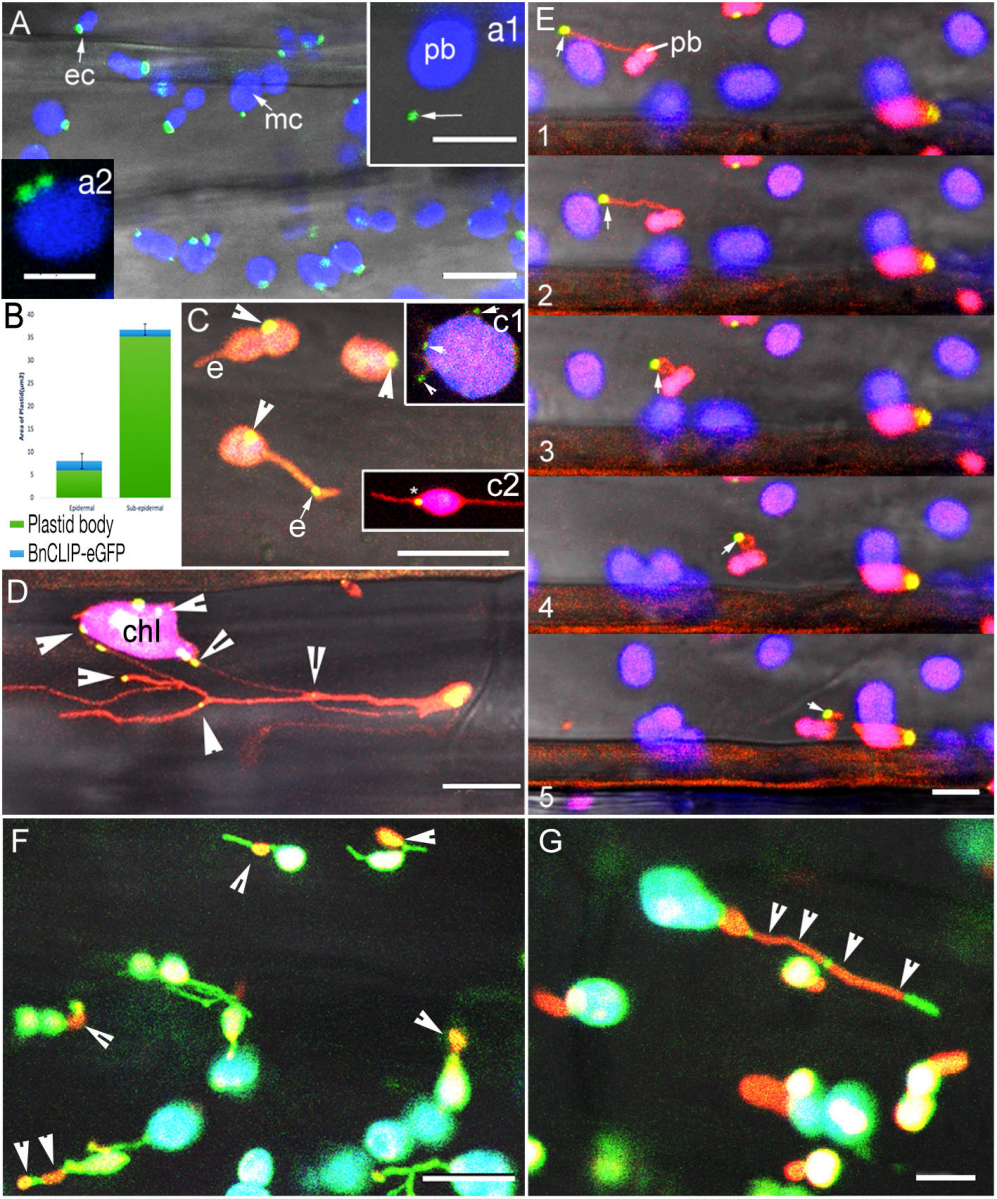
Characteristic localization patterns for BnCLIP1-eGFP and two IEM localized sugar-phosphate/phosphate transporters in hypocotyl cells in stable transgenic plants. **(A)**. BnCLIP1-eGFP (green) patches on the chloroplast envelope in hypocotyl epidermal (ec) and larger chloroplasts in subtending layers (mc). Chlorophyll autofluorescence is false coloured blue. (a1 – arrow shows a BnCLIP1-eGFP puncta ca. 3µm away from the plastid body (pb); a2 – large patches can be resolved into smaller, closely localized punctae). (**Supplementary Movie 1**). **(B)**. A bar graph depicting the mean area of BnCLIP1-eGFP puncta relative to the total plastid area for epidermal and sub- epidermal chloroplasts (n=27 for each chloroplast type). Standard Deviation of BnCLIP1-eGFP enriched area for epidermal and sub-epidermal chloroplasts is 1.65 and 1.2µm^2^, respectively. **(C)**. BnCLIP1-eGFP patches exhibited a stochastic distribution on plastid body (arrowheads) and on stromules (e). (c1 - multiple punctae (arrowheads) suggestive of positive correlation with place of stromule formation; c2 – plastid with two stromules, one of which (*) is completely opposite the BnCLIP1-eGFP puncta. (**Supplementary Movie 2**). **(D)**. Double transgenic lines for BnCLIP1-GFP and photoconverted tpFNR-mEosFP in the *arc6* mutant background reinforced the stochastic localization of the multiple punctae (arrowheads) on the large chloroplast body (chl) and stromules. **(E)**. Representative images (panels 1-5) showing the dynamic behaviour of a stromule and the consequent change in spatial relationship between BnCLIP1-eGFP patches (arrows) and the plastid body (pb). Note that the size of BnCLIP1-eGFP did not change on an extended stromule (panels 1, 2). **(F)**. Control double transgenic plants expressing GPT1-mEosFP and stroma targeted tpFNR-EGFP showed orange-red IEM localized patches of GPT1-mEosFP (arrowheads) that appear very similar to BnCLIP1-eGFP punctae on stromules shown in **(B, E)**. Similar punctae were observed for the second control TPT1-mEosFP. **(G)**. Compared to the discrete BnCLIP1-eGFP punctae being maintained during dynamic behaviour of a stromule **(E)** the TPT1-mEosFP patches stretch over long regions (arrowheads) in extended stromules. Similar expanded localization was observed for GPT1-mEosFP punctae. Stromule fluorescence green in panels **(F, G)**; chlorophyll autofluorescence depicted in blue; Overlay of red, green and blue in some chloroplasts shows up as white colour. Size bars – **(A)** =10, a1 = 5, a2 = 2.5; **(C)**, c1, c2, **(D, E)** = 10; **(F, G)** = 5 µm.

In agreement with the transient expression-based experiments of Tan et al. (2011) observations on soil grown, 8-10 days old seedling from 5 independent stable transgenic lines of BnCLIP1-eGFP in *Arabidopsis thaliana* showed 1 to 4 patches of 400 ± 250 nm on all chloroplasts (**Figure 1A**; n = 350 chloroplasts). Both, profile and face-on views in cotyledon, hypocotyl and young leaves strongly suggested that several BnCLIP1-eGFP punctae clustered together to produce a large polar patch in the small (long axis 2.5 - 3µm) epidermal cell chloroplasts (Barton et al., 2018; PCC, **Figure 1A-ec**; **Figure 1A2**). In contrast, although punctae size remained similar in the relatively large chloroplasts (5-8 µm diameter) in sub-epidermal layers (MCC), they were distributed randomly over the chloroplast surface. Analysis of mean area of BnCLIP1-eGFP puncta relative to the total plastid area for epidermal (mean area 6.06 µm^2^) and sub-epidermal chloroplasts (mean area 35.37µm^2^) using 27 plastids for each category reinforced the pronounced disparity in the localization of BnCLIP1-eGFP puncta between them (**Figure 1B**). In comparison to sub-epidermal plastids, BnCLIP1-eGFP puncta occupy a greater proportion of the total area in epidermal chloroplasts (4% in sub-epidermal compared to 34% in epidermal chloroplasts). While there was significant difference in the distribution pattern of BnCLIP1-eGFP punctae in these two chloroplast types in young plants, as also reported by Tan et al. (2011), large chloroplasts in senescing cotyledons in 15-20 days old plants could exhibit up to 12 discrete punctae of variable sizes.

A difference in punctae localization was also observed in BnCLIP1-eGFP plants grown on ½ MS (Murashige and Skoog, 1962) medium supplemented with 0.75% sucrose. Up to 45% PCC (n=200 chloroplasts) had BnCLIP1-eGFP punctae located at distances ranging from 3 to 8 µm from the main plastid body (e.g. **Figure 1A**). Although Tan et al., 2011 had reported a cytoplasmic localization for BnCLIP1-eGFP (pb -**Figure 1A** arrow) they had not investigated it further. Our investigations focused next on this aspect of BnCLIP1-GFP localization.

### BnCLIP1-eGFP punctae observed away from the plastid body are present at stochastic locations along stromules

Sporadically, the envelope of all plastids is extended to form thin tubules (Wildman et al., 1962) called stromules (Köhler and Hanson, 2000). Stromules have been suggested to interact with small organelles such as peroxisomes and mitochondria whose normal diameters range from 1 to 1.5 µm (Kwok and Hanson, 2003; Kwok and Hanson, 2004). Using double transgenic plants of BnCLIP1-eGFP with YFP-SKL (peroxisomal marker; Mathur et al., 2002), and BnCLIP1-eGFP and mito-mEosFP (Mathur et al., 2010) the BnCLIP1-eGFP punctae located far away from the plastid body were not found to colocalize with either peroxisomes or mitochondria (results not shown).

Therefore, we considered the possibility that the cytoplasmic localization of BnCLIP1-eGFP may reflect the presence of punctae directly on stromules that had been extended away from the plastid body. Double transgenic plants expressing a green to red photoconvertible tpFNR-mEosFP (Schattat et al., 2012) along with BnCLIP1-eGFP were created. Following a 5 second exposure to a violet-blue light for photo-converting mEosFP the stroma fluoresced orange-red while BnCLIP1-eGFP punctae remained green (**Figure 1C**). This confirmed that BnCLIP1-eGFP localization far from the plastid body was due to its presence on an extended stromule. Comparisons between control plants expressing the stroma marker tpFNR-EGFP (Schattat et al., 2011a) and F3 generation tpFNR-mEosFP-BnCLIP1-eGFP did not reveal an increase in the number of plastid extensions or BnCLIP1-eGFP patches. This suggested that use of a strong, constitutively active Cauliflower mosaic virus 35S promoter to achieve a high expression of mEosFP and BnCLIP1-eGFP affects neither stromule formation or the BnCLIP1-eGFP localization pattern.

Whether BnCLIP1-eGFP punctae on stromules exhibit a specific positional correlation with the main plastid body was assessed next. Stable transgenic plants expressing inner envelope membrane localized photoconvertible mEosFP fusion with GLUCOSE 6-PHOSPHATE/PHOSPHATE TRANSLOCATOR1 (GPT1; Fliege et al., 1978) and TRIOSE PHOSPHATE/PHOSPHATE TRANSPORTER1 (TPT1; Flügge, 1999) were reported earlier (Delfosse et al., 2018) and served as controls (**Figure 1F**). Patches of varying sizes, similar to those observed for BnCLIP1-eGFP were observed for both transporters. Similar to that concluded for the transporters (Delfosse et al., 2018) BnCLIP1-eGFP localization could occur on any part of a stromule and was considered stochastic **Figure 1C**).

Nevertheless, since BnCLIP1-eGFP patches were occasionally observed on small protuberances of the chloroplast envelope (**Figure 1C-c1**), a possible correlation between the lipase enriched region of a chloroplast and the origin site of a stromule was assessed. Observations showed that 68% (n = 150 chloroplasts) of the stromules formed in regions away from BnCLIP1-eGFP punctae (**Figure 1C-c2**). The stochastic localization of BnCLIP1-eGFP was tested further in the Arabidopsis *accumulation and replication of chloroplasts 6* (*arc6*) mutant with greatly enlarged mesophyll chloroplasts (Pyke et al., 1994) and more frequent, long stromules (Holzinger et al., 2008) (**Figure 1D**). As compared to the non-mutant controls, the overall number of BnCLIP1-eGFP punctae on the plastid body, as well as those that localized to stromules increased in the *arc6* plastids with stroma-targeted tpFNR-mEosFP and maintained a stochastic distribution. Using time-lapse imaging it was determined that the different patches present on extended stromules in *arc6* mutant (**Figure 1D**) and in double transgenic plants expressing tp-FNR-mEosFP and BnCLIP1-eGFP (**Figure 1E** panels 1-5; **Supplementary Movie 2**) could move away or towards the plastid body, according to the extension or retraction of the tubule. Control plants of GPT1-mEosFP showed punctae (**Figure 1F**) similar to the ones from BnCLIP1-eGFP. However, clear differences in behaviour became apparent between the punctae in control plants expressing GPT1mEosFP (**Figure 1F**) and TPT1-mEosFP (**Figure 1G**) and BnCLIP1-eGFP punctae (**Figure 1E**) in extended stromules (**Figure 1E** versus **Figure 1G**). While BnCLIP1-eGFP punctae maintained their small size (**Figure 1E**; **Supplementary Movie 2**) the punctae in TPT1-mEosFP (**Figure 1G**) and the second control plants of GPT1-mEosFP (**Figure 1F**); Delfosse et al., 2018) extended considerably to cover large regions of a stromule (arrowheads in **Figure 1G**).

Although limited to transient expression assays in tobacco leaves, a major finding by Tan et al. (2011) is the demonstration that BnCLIP1-eGFP punctae co-localize with the ER. Our subsequent investigations therefore focused on whether BnCLIP1-eGFP can be used as a reliable fiducial in stable transgenic lines of Arabidopsis for observing plastid interactivity with the ER and its consequences.

### BnCLIP1-eGFP enriched regions on the envelope delineate ER-interacting locations

**Figure 2:**
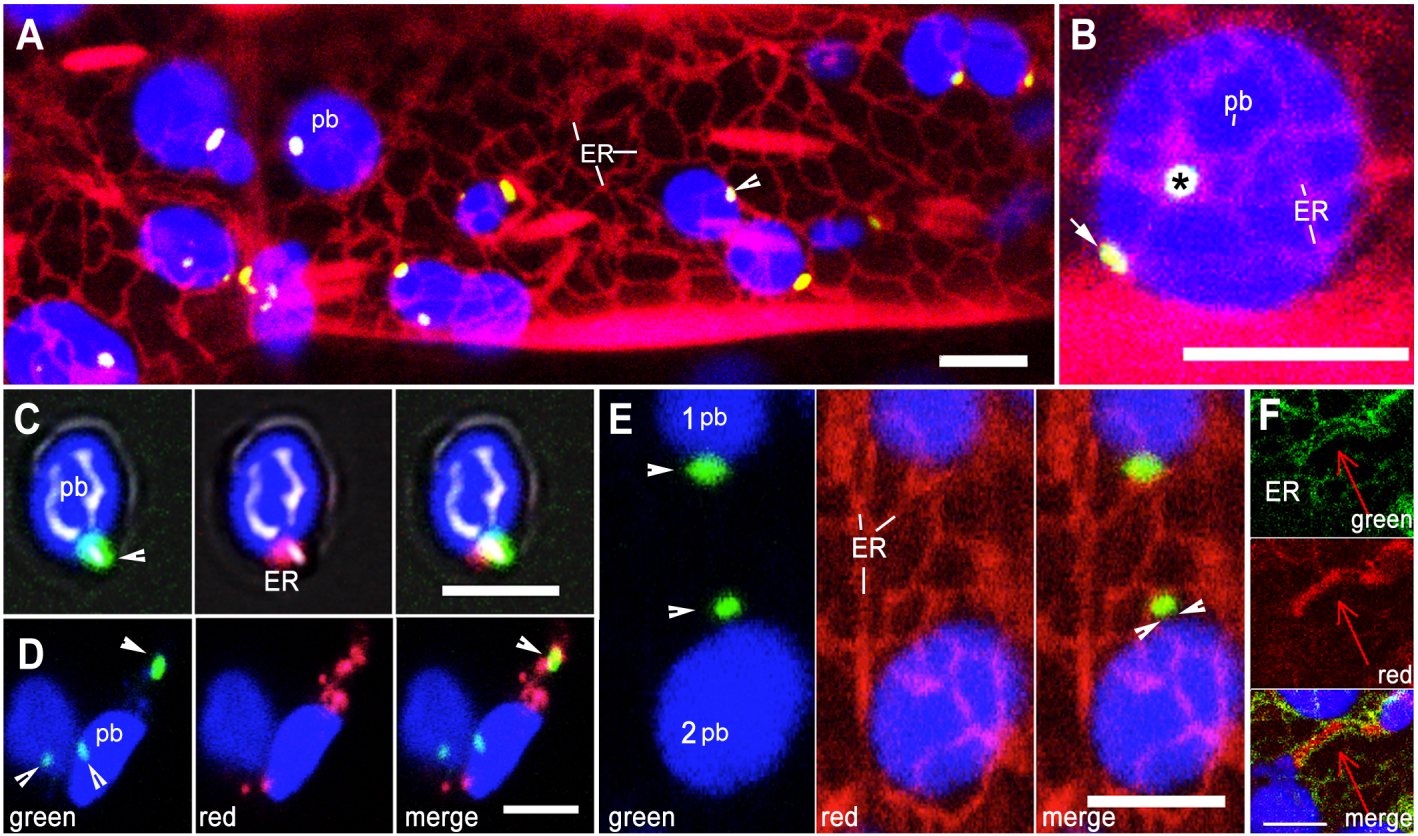
Relationship between envelope localized BnCLIP1-eGFP punctae and the ER. **(A)**. Representative image from amongst 350 hypocotyl cells in a double transgenic Arabidopsis line expressing ER lumen-retained mRFP (red) and BnCLIP1-eGFP punctae (green; arrowhead) against the backdrop of chlorophyll autofluorescence (blue plastid body -pb). **(B)**. Representative image chosen from a snapshot amongst 10 time-lapse movies that shows both face-on (asterisk) and profile (arrow) views indicating close alignment of BnCLIP1-eGFP punctae and ER at two locations on a sub-epidermal chloroplast (blue; pb). Based on **Supplementary Movie 3** averaged Pearson’s coefficient of 0.93 and Manders’ overlap coefficient of 0.947 were obtained for the region marked by ‘*’. **(C)**. A green BnCLIP1-eGFP puncta (arrowhead) on an isolated chloroplast (blue-pb) colocalizing with red fluorescent ER (merged image). The averaged Manders’ overlap coefficient for RER to BnCLIP1-eGFP was 0.81. **(D)**. Two isolated chloroplasts (blue; pb) with three BnCLIP1-eGFP punctae (arrowheads) and red fluorescent ER. The merged image shows only one BnCLIP1-eGFP puncta (arrow), located at a distance from the plastid body (pb) enmeshed in the ER (merged image) while the remaining punctae at the bottom of the panels do not colocalize with ER. Average Pearson’s coefficient of 0.718 indicates a high degree of colocalization. **(E)**. Simultaneous visualization of ER tubules and BnCLIP1-eGFP punctae in two chloroplasts show that although BnCLIP1-eGFP puncta may appear close to the plastid body (1- pb) or slightly distanced from it (2 pb; facing arrowheads) only a small point of interactivity with the ER is maintained. **(F)**. Representative image showing ER (green) around a large TPT1-mEosFP enriched region of a tubular plastid extension contrasts with the small, punctate ER-interaction point created by BnCLIP1-eGFP. Blue=Chlorophyll autofluorescence, Green=BnCLIP1-eGFP punctae, Red= mRFP-HDEL tubules. Size bars: =5µm.

A loose cage of continuously rearranging ER membranes around plastids creates numerous possibilities for transient contacts between the two organelles (Schattat et al., 2011a; Kumar et al., 2018; Mathur, 2021; Mathur et al., 2022). A double transgenic Arabidopsis line expressing BnCLIP1-eGFP and an ER-lumen retained RFP-HDEL (Sinclair et al., 2009) was generated and used along with a control, double transgenic line expressing TPT1-mEosFP (Delfosse et al., 2018) and ss-eGFP-HDEL (Haseloff et al., 1997). Simultaneous observations on BnCLIP1-eGFP patches and the ER were made in hypocotyl cells in 8-10-day old seedlings grown overnight in the dark. Time-lapse observations taken on 10 cells observed from each of 35 plants (350 cells total) showed continuously rearranging ER tubules, 76% of which were found within 500 nm of the BnCLIP1-eGFP enriched punctae (**Figure 2A, B**). A strong and persistent association between RER and BnCLIP1-eGFP punctae across multiple frames was also indicated by Manders’ overlap coefficient analysis (Manders et al., 1993). The full encasement of BnCLIP1-eGFP puncta within the endoplasmic reticulum (ER) would result in Manders’ coefficient of 1. Based on five consecutive frames from Supporting **Movie 3** that formed the basis for **Figure 2B** the averaged Manders’ coefficient for RER to BnCLIP1-eGFP was found to be 0.947.

Whether, as suggested by the laser-based ER pulling experiments of Andersson et al. (2007), the apparent plastid-ER interactivity resulted in connectivity between the organelles, was tested by isolating chloroplasts from the BnCLIP1-eGFP + RFP-ER line. Five independent isolations showed a variable mix of chloroplasts between the experiments where they fell into categories with no fluorescent signal, green BnCLIP1-eGFP punctae alone, red fluorescent ER patches alone, separately localized BnCLIP1-eGFP and ER punctae, and ER patches located on BnCLIP1-eGFP enriched regions (**Figure 2C, D**). Compared to the majority of chloroplasts with BnCLIP1-eGFP showing proximity to RFP-ER in living cells (**Figure 2A, B**) less than 25% isolated chloroplasts showed ER at exactly the same location as BnCLIP1-eGFP. Although the low number of chloroplasts maintaining BnCLIP1-eGFP-RER colocalization reinforced the idea of plastid-ER interactivity the observations also raised the possibility that the isolation of chloroplasts from their native subcellular environment changed organelle membrane properties and interactivity. Alternatively, our preparations could contain insufficient number of chloroplasts with an intact envelope.

In independent observations on intact cells (n=100), small BnCLIP1-eGFP enriched punctae localized at a distance from the plastid body while remaining attached to the ER (**Figure 2E**) suggesting that the BnCLIP1-eGFP punctae served as the main interaction points between the two organelles. By contrast, rather than ER-interacting punctae, control double transgenic TPT1-mEosFP + GFP-ER plants showed long ER-lined channels filled with the fluorescent fusion protein (**Figure 2F**).

Whether BnCLIP1-eGFP punctae and ER remain associated during plastid displacement was investigated next.

### Interactivity with ER is maintained during movement of small chloroplasts

**Figure 3:**
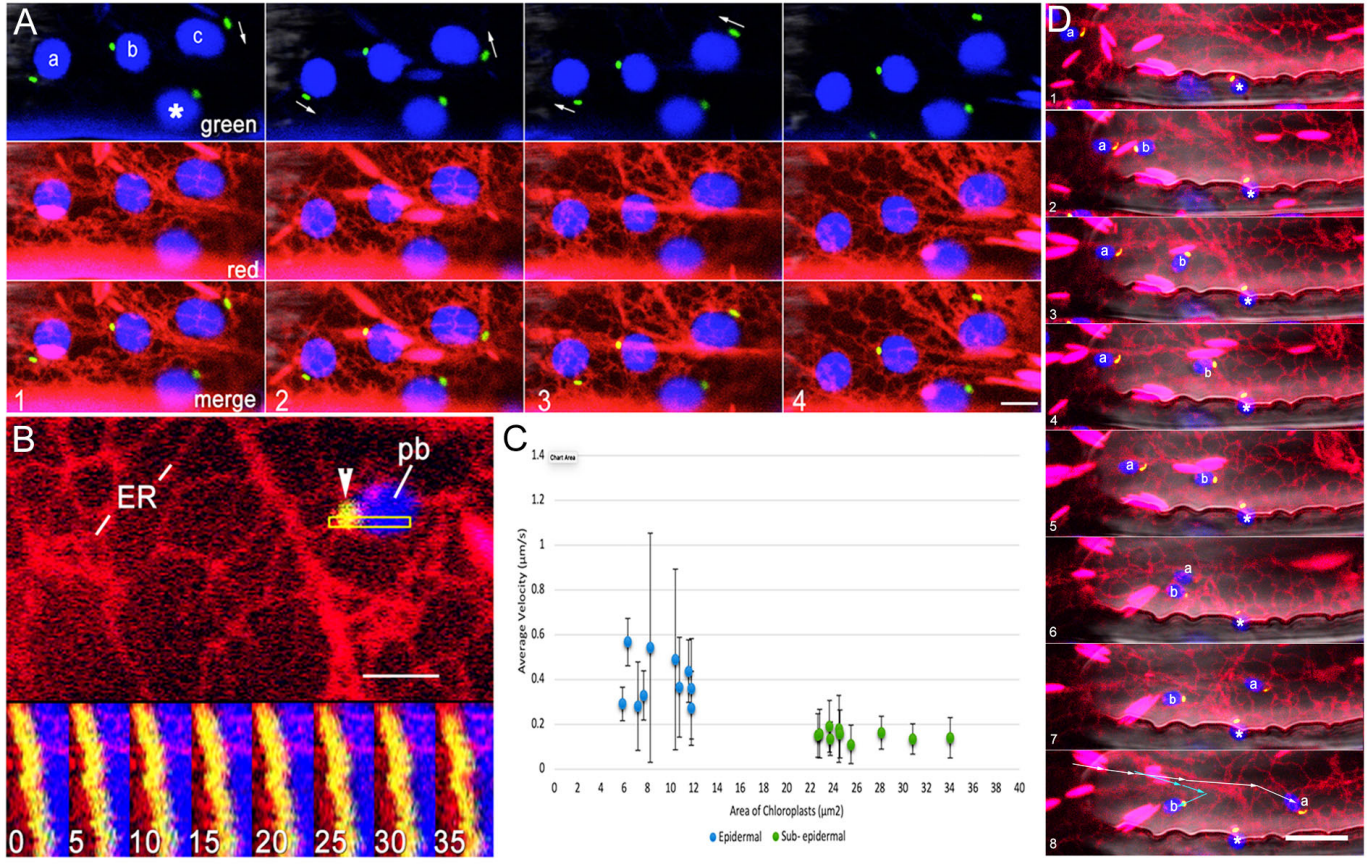
Association between BnCLIP1-eGFP enriched regions of the envelope and the ER is maintained during plastid displacement. **(A)**. Four consecutive snapshots from a representative (n=10 x,y,t series) 60 second time-lapse sequence show the independent movements of 4 epidermal chloroplasts in association with ER tubules. Arrows in the four panels depict variations in the behaviour of plastids labelled (a-c) while the motion of plastid ‘ * ‘ remains relatively limited. ER tubules appear to maintain continuous association with BnCLIP1-eGFP puncta on each plastid. **(B)**. A kymograph establishing continuous spatiotemporal association between the ER and BnCLIP1-eGFP enriched puncta in the region of interest shown by outlined rectangle in top portion of the figure. Each of the frames, numbered 1 to 8, represents the fifth consecutive frame from a total of 35 frames spanning 1.27 minutes of a time-lapse (x,y,t) series. **(C)**. Graphical representation of the average velocity of epidermal and sub-epidermal chloroplasts relative to their area (n=10 for each chloroplast type). Mean epidermal chloroplast velocities in µm/s = 0.39 ± 0.15 (standard error) and 0.22 ± 0.10 for sub- epidermal chloroplasts. As depicted by the graph the average velocity of epidermal chloroplasts surpasses that of sub- epidermal chloroplasts. Increased variability in velocity among epidermal chloroplasts, is indicated by the extended error bars and underscores their heightened dynamic behavior within plant cells. **(D)**. Eight sequential images from a time-lapse series spanning 200 seconds (**Supplementary Movie 4**) show polar localization of BnCLIP1-eGFP (green) at the leading pole of 3 small chloroplasts (blue) labelled ‘a’ ‘b’ and ‘*’. The BnCLIP1-eGFP enriched region maintains close connectivity with the ER continuously as the chloroplasts are displaced at different rates over different distances (summarized by arrows in panel 8) within the cell. During their movement chloroplasts ‘**a**’ and ‘**b**’ maintain averaged Pearson’s correlation coefficient of 0.603 and 0.678, respectively. Notably plastid ‘*’ remains confined within a small area. Scale bars **(B)** =2.5; **(A)**= 5; **(D)** = 10µm.

Small epidermal chloroplasts in Arabidopsis exhibit movement (Barton et al., 2018). A double transgenic line expressing BnCLIP1-eGFP and RER was used for short time-lapse movies. Single sequential frames from the time-lapse series (**Figure 3A**) were used to build kymographs (**Figure 3B**) to ascertain spatiotemporal interactivity between chloroplasts with BnCLIP1-eGFP punctae and the ER. While a high Pearson’s correlation coefficient averaging 0.675 supported BnCLIP-eGFP and RER colocalization it also became apparent during our observations that the larger sub-epidermal chloroplasts had less displacement than small epidermal ones. Indeed, estimating the average velocities of small epidermal chloroplasts versus those of larger sub-epidermal chloroplasts confirmed that small chloroplasts showed greater displacement over time with average velocities of 0.39 ± 0.15 (S.E.; n = 10). By comparison sub-epidermal chloroplasts maintained average velocities of 0.22 ± 0.10 (S.E.; n=10) and were more likely to oscillate and show small displacements (**Figure 3C**). As shown in **Figure 3D** the movement of small plastids was asynchronous and erratic. The movement appeared to accompany changes in ER organization around the BnCLIP1-eGFP enriched pole of the chloroplasts and suggested a direct involvement of ER association in the process.

A second phenomenon displayed readily by small chloroplasts is their extension of stromules (Barton et al., 2018). The role that ER associated with a BnCLIP1-EGFP punctae may have in directing stromule dynamics was assessed next.

### Dynamic behaviour of plastid extensions and ER occur in tandem

Based on our observations (**Figure 1**), BnCLIP1-eGFP overexpression does not lead to stromule formation. However, given the interactivity that is maintained between BnCLIP1-eGFP punctae and contiguous ER (**Figure 2**, **3**), the presence of a puncta on a stromule that is already extended along a cytoskeletal element (Erickson et al., 2018; Kumar et al., 2018) links plastid-ER behaviour at that point. Consequently, ER reorganization, also occurring along cytoskeletal elements, results in tandem stromule reorganization too. It would be expected that when attachment between the two organelles breaks, a stromule would retract towards the plastid body.

**Figure 4:**
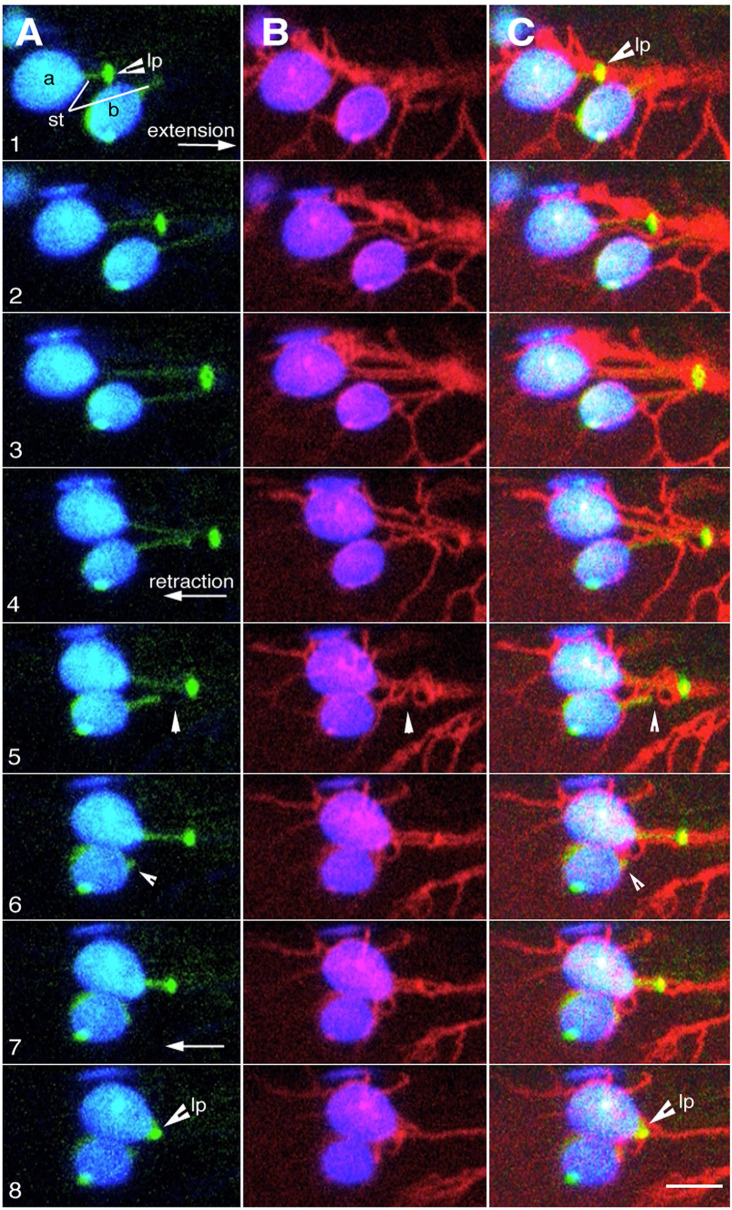
Dynamic behaviour of plastid extensions is due to BnCLIP1-eGFP enriched regions interacting with contiguous ER. **(A–C)**. Eight sequential snapshots taken from an x,y,t time-lapse series spanning 300 seconds (based on **Supplementary Movie 5**) shows ER (red) association with lipase BnCLIP1-eGFP enriched region (lp) of two plastids (a, b) during the extension (panels 1-3) and retraction of a stroma targeted YFP highlighted tubular stromule (st). Quick retraction of the stromule to the plastid body, observed in panels 4 to 8 is a result of break in connectivity between the BnCLIP1-eGFP enriched region and the ER (arrowhead pointing to breakpoint– panel 5). Size bar = 5 µm.

**Figure 5:**
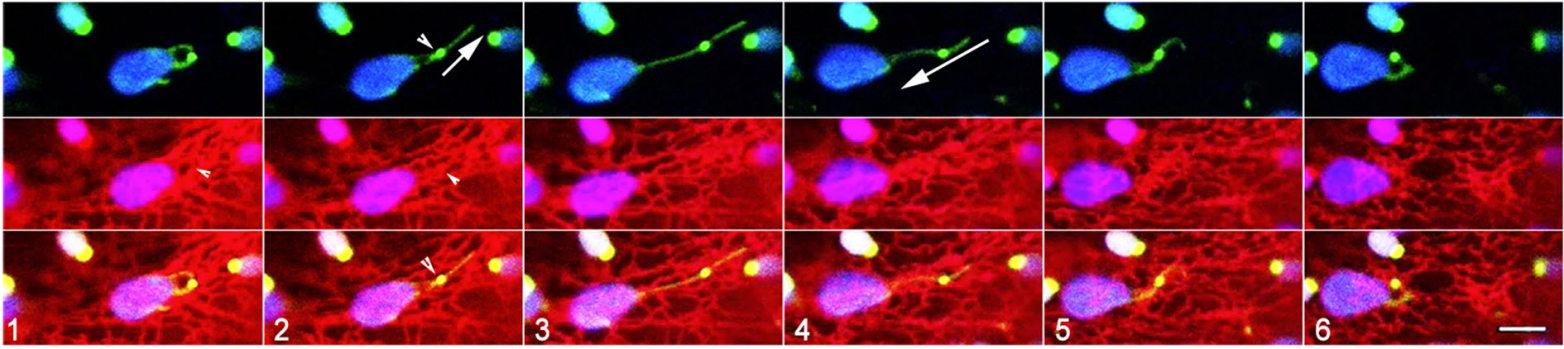
Suggested dynamic behaviour of plastid extensions and associated ER. Six sequential snapshots taken from **Supplementary Movie 6** that spanned 300 seconds establish that subtle alterations in the shape of a stromule may result from very localized interactions with the ER while another portion remains unaffected. The region with BnCLIP1-eGFP extends (panel 1-4), retracts (panels 5-8), and morphs into hooked forms that correlate with dynamic reorganization of underlying ER. The tip portion, without BnCLIP1-eGFP remains extended in the frames 1-6 and is perhaps due to independent interactions with cytoskeletal elements. Size bar = 5 µm.

Using a triple transgenic line expressing BnCLIP1-eGFP, RFP-HDEL and a stroma-targeted YFP 6 time-lapse sequences ranging from 3-5 minutes were recorded of instances where BnCLIP1-eGFP punctae located on extended stromules associated with ER. As shown in **Figure 4** (**Supplementary Movie 5**) an extended stromule was pulled outwards from the plastid body and retracted to it upon break of connectivity with the ER (**Supplementary Movie 5**). By contrast, **Figure 5** depicts that the BnCLIP1-eGFP puncta may not always be located at the tip of a stromule. The subsequent stromule behaviour resulting in variable shapes (**Figure 5**; **Supplementary Movie 6**) may be attributed to only a small region of the stromule being affected by contiguous ER behaviour. It is possible that a portion of the stromule far from the ER-interacting BnCLIP1-eGFP puncta remains unaffected by neighbouring-ER dynamics while maintaining its alignment with a cytoskeletal element.

Presently, the lack of a reference point for both plastids and the ER or an ER-localized fiducial precludes quantitative assessment of dynamics of the linked organelles. However, observations of stromule shape modulation, extension and retraction do reinforce the idea of interactivity between a small region of a chloroplast and the surrounding ER.

## Discussion

Although laser-based experiments have established strong connectivity between the ER and chloroplasts (Andersson et al. (2007), the relatively large plastid surface and the thin and dynamic ER tubules, make it difficult to identify a specific region of plastid-ER interaction. The limited number of BnCLIP1-eGFP enriched spots on each plastid narrowed down the regions for observations and allowed us to observe localized plastid-ER interactivity and its consequences. Although observed only through transient expression assays Kumar et al. (2018) have strongly suggested that stromule behaviour may involve intimate alignment and interaction between the ER and cytoskeletal elements. Moreover, earlier observations using transgenic plants have clearly established the presence of a loose, continuously reorganizing ER cage around every plastid and demonstrated that the branching of plastid stromules occurs through their alignment with ER tubules (Schattat et al., 2011a; Schattat et al., 2011b). Though observations on the tandem behaviour of plastid extensions and contiguous ER resulted in speculations about the presence of MCS between the two organelles (Wang and Benning, 2012; Pérez-Sancho et al., 2015), actual demonstration of membrane connectivity and its consequences had been missing so far.

The availability of BnCLIP1-eGFP, a putative lipase that localizes to the chloroplast envelope and demonstration of its interactivity with the neighbouring ER (Tan et al., 2011) provided a useful tool to exploring the plastid-ER relationship further. Notably, BnCLIP1 has been considered a putative triacyl glycerol (TAG) lipase with homology to At1g06800, another putative TAG lipase gene from *Arabidopsis* (Tan et al. (2011). Since demonstration of interactivity between BnCLIP1-eGFP punctae and ER was carried out only through transient overexpression assays in agroinfiltrated tobacco leaves, a clear estimation of its lipase activity in tobacco has been missing (Tan et al., 2011). Nevertheless, subsequent publications have treated BnCLIP1 as a general TAG lipase and speculated about its role as a membrane contact site (MCS) promoting protein (Block and Jouhet, 2015; Mueller-Schuessele and Michaud, 2018; Michaud and Jouhet, 2019; Li et al., 2020; Leterme and Michaud, 2022). Whether the Arabidopsis lines overexpressing BnCLIP1 generated by us have an overall increase in lipase activity has not been investigated. However, the transgenic lines used for our observations are phenotypically similar to wild-type Arabidopsis plants (Columbia ecotype), flower and produce normal seeds regularly. Most important, other than showing BnCLIP1-eGFP enriched punctae, plastid shapes and behaviour have remained unchanged in these BnCLIP1-eGFP overexpressing Arabidopsis lines. We speculate that under our conditions, the localization of this non-native lipase to a few, very small regions of the chloroplast envelope, has minimized any effects that may occur on the overall growth and development of the transgenic Arabidopsis. Since several other lipases from Arabidopsis have been shown to have similar punctate localization on chloroplasts (Seo et al., 2009), further investigations using them may reveal scorable changes in Arabidopsis due to increased lipase activity. However, in complete concurrence with the report of Tan et al. (2011), BnCLIP1-eGFP enriched regions of chloroplasts were observed interacting with contiguous ER (**Figures 2**–**4**, **Supplementary Movies 3**, **4**, **5**, **6**). Notably, transgenic plants overexpressing other proteins with punctae localizations similar to BnCLIP1-eGFP, used as controls, did not exhibit similar patterns of interactivity with the ER.

Earlier studies have established that both chloroplasts and the ER rely upon the actin cytoskeleton and associated motors for their dynamic behaviour (Ueda et al., 2010; Cao et al., 2016; Wang et al., 2017). Our observations now demonstrate that while maintaining their respective relationship to cytoskeletal elements, plastids and the ER also link together transiently. As shown here, the association results in their tandem behaviour. Interestingly, dissection of chloroplast displacement revealed that unlike small epidermal chloroplasts that show greater velocities and large displacements, large chloroplasts are slow to move (**Figure 3C**). Instead they tend to oscillate and rotate until drawn into bulk cytoplasmic rearrangements occurring in response to major stresses induced by light or wounding. Considering that ER-interacting BncLIP1-eGFP punctae are more dispersed over the surface of large plastids (**Figure 1B**) their low displacement may be attributed to their being pulled in several different directions by the associated ER strands.

Our observations provide strong support for the idea that the dynamic behaviour of individual chloroplasts, reflected in their tubular extensions, is dependent on a pulling force applied to the plastid envelope (Erickson and Schattat, 2018) possibly through the presence of MCS between plastids and the ER (Schattat et al., 2011b; Wang and Benning, 2012). In concurrence with the work of Kumar et al. (2018) both extension and retraction may involve intimate interactions of ER with cytoskeletal elements.

As already concluded by Tan et al. (2011) and reinforced by our observations, the linked transient behaviour of chloroplasts and the ER suggests membrane linkage. Consequently, BnCLIP1 may be considered to be a MCS facilitating protein. A key requirement for MCS formation is the presence of a membrane resident protein or protein-complex at the site. While such proteins may have other functions also, their presence facilitates increased proximity between the partner organelles (Prinz, 2014; Bohnert, 2020; Prinz et al., 2020). Studies using different MCS proteins have also suggested that the motor protein-based movement of an organelle can alter the positioning of a connected organelle (Knoblach et al., 2013; Valm et al., 2017; Guo et al., 2018). BnCLIP1-eGFP does fulfil these requirements.

**Figure 6:**
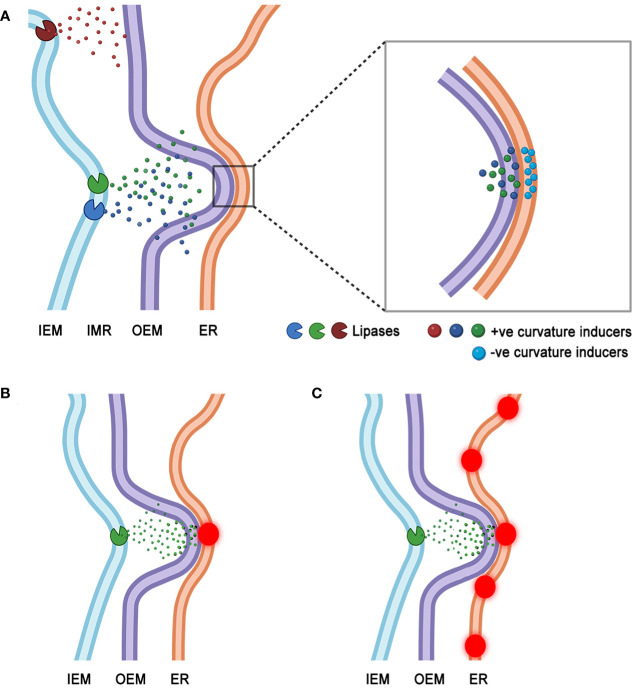
A speculative model for MCS formation that considers lipase induced alterations in membrane topology leading to interactions between positively curved region of the plastid envelope and a negatively curved, concave region of a juxtaposed ER membrane. **(A)**. Activity of plastid inner envelope membrane (IEM) localized lipases releases positive curvature-inducing lyso-lipids and free fatty acids, that could accumulate in the inter-membrane region (IMR) of the envelope. Eventual transit of the HI type acyl-moieties through the plastid outer envelope membrane (OEM) may induce tiny protuberances in it. The OEM protuberances may interact with apposing regions of negative curvature on ER membranes. Such concavities may be formed transiently due to the presence of HII phase promoting acyl-lipid moieties such as diacylglycerol (DAG) and phosphatic acid (PA). While transient MCS (zoomed in region) may form at juxtaposed +ve and -ve curved regions of the two organelles they may be further stabilized by membrane bending and cytoskeletal interacting proteins recruited to the site. **(B)**. MCS may develop at a single point of interaction (red puncta) created by +ve curvature-causing moieties on the OEM and a single -ve curvature region on the ER membrane. **(C)**. The dynamic reorganization of ER may allow several regions of -ve curvature to interact with a single region of +ve curvature on the plastid OEM. A series of transient MCS (red punctae) may result between the two organelles.

At present a clear demonstration of localized and very specific lipase activity at BnCLIP1-eGFP enriched regions of the plastids has not been possible. It is quite possible that BnCLIP1-eGFP punctae result from overexpression of a non-native protein and are prone to non-specific oligomerization artifacts known from GFP overexpression (Costantini et al., 2012). A combination of correlative electron microscopy and confocal microscopy techniques is required and being pursued. However, our present findings allow a speculative model to be presented wherein localized lipase activity may introduce alterations in membrane topology to foster MCS formation. It is well established that lipase activity generates free fatty acids (FFA) and lyso lipids (Andersson et al., 2004; Ryu, 2004; Lee and Park, 2019). Such small molecules can directly affect membrane topology through positive curvature-induced bilayer distortion (Farsad and De Camilli, 2003; Arouri and Mouritsen, 2013; Jouhet, 2013; Fan et al., 2017). Consequently, the model (**Figure 6**) suggests that accumulation of FFA and lyso-lipid products due to lipase activity leads to one or more small regions of the plastid envelope membrane being distorted to form tiny protuberances. MCS may result when a protruding region connects with a complementary concave region on contiguous, extraplastidial membranes. Indeed, regional accumulations of small cone shaped acyl-lipids such as phosphatidic acid (PA) and diacylglycerol (DAG) are known to modulate ER membrane topology by creating transient regions of negative curvature and small concavities (Chernomordik, 1996; Burger, 2002).

The suggested model does not exclude the recruitment of additional proteins to topologically altered regions of the membranes. These proteins may themselves have membrane bending functions that further tighten the attachment to sustain MCS for longer durations (Breeze et al., 2016; Wang et al., 2016). Since both plastids and ER have been shown to depend upon cytoskeletal elements and associated motor proteins for their movement and behaviour (Ueda et al., 2010; Cao et al., 2016; Wang et al., 2017; Erickson et al., 2018; Kumar et al., 2018; Pain et al., 2023) it is important to consider that such interactions would play important roles in both the formation and breaking of membrane contacts.

Further experiments investigating different PLA1 lipases such as DONGLE, DAD1 and DAD-like lipase 2 (Ishiguro et al., 2001; Turner et al., 2002; Hyun et al., 2008; Seo et al., 2009; Ellinger et al., 2010) from Arabidopsis are being used to assess the lipase mediated model for MCS formation and will be reported separately.

## Materials and methods

### Fluorescent fusion protein constructs

BnCLIP1-eGFP (Tan et al., 2011) was used as provided. The eGFP4-ER (Haseloff et al., 1997, mRFP-HDEL (Sinclair et al., 2009), tpFNR-EGFP (Marques et al., 2003; Schattat et al., 2011a), tpFNR-mEosFP (Schattat et al., 2012), GPT-EGFP and TPT1-mEosFP (Delfosse et al., 2018) used for generating stable transgenic lines in Arabidopsis have been reported earlier.

### Transient expression in plants

Transient expression was used only for checking different fusion protein constructs before they were used for stable transformation of Arabidopsis. All DNA constructs were checked for expression using *Agrobacterium tumefaciens* (strain GV3101::pMP90) infiltration of tobacco leaves. For agroinfiltration the bacteria were suspended in Agrobacterium infiltration media (10 mM MgCl2, 5 mM MES, pH 5.3, 150 mM acetosyringone), and incubated at room temperature until an optical density of 0.8 at 600 nm was reached. Simultaneous introduction of two agrobacteria expressing different genes involved mixing the two bacterial suspensions in equal quantities before injecting them into leaves of 4-week old *Nicotiana benthamiana* plants using a needle-less syringe. Leaf pieces of ca. 4.5 cm^2^ were excised at around 32 hours after infiltration and viewed under a fluorescence microscope.

### Transgenic plants and growth conditions

Single and double transgenic lines of *Arabidopsis thaliana* (Columbia ecotype) were created through *Agrobacterium tumefaciens* mediated floral dip transformation (Clough and Bent, 1998) or through crossing of already selected stable transgenic lines. Seeds for transgenic line ‘pt-yk’ (CS16267; Nelson et al., 2007) expressing a stroma targeted YFP were obtained from ABRC and used for creating a triple transgenic line with BnCLIP1-eGFP and mRFP-ER (Sinclair et al., 2009). All seeds of Arabidopsis were stratified for 2 days at 4°C. For soil-based experiments plants were grown on Sunshine mix LA4 (Sun Gro Horticulture, USA) soil in sealed Magenta boxes. Alternatively, plants were grown in plastic petri dishes containing ½ concentration of Murashige and Skoog basal medium (1962) with B5 vitamins (Sigma M0404), 0.75% sucrose and 3g/L phytagel (Sigma-Aldrich) (pH adjusted to 5.8 before autoclaving). Plants were grown for 7–14 days under a long-day light regime (16-h-light–8-h- dark) under light intensity of 120 ± 20 μmol m^−2^ s^−1^ light and ambient temperature of 21 ± 2°C.

### Chloroplast isolation

A small-scale chloroplast isolation method based on Salvi et al. (2011) was followed. Entire, 3-week-old plants were cut and dispersed in 5 mL of homogenization buffer and filtered through six layers of cheesecloth into a small beaker. The tissue remaining in the cheesecloth was resuspended in 5 mL of homogenization buffer, filtered twice through the cheesecloth before pelleting the filtrate at 1000 g for 5 min using a swinging bucket rotor centrifuge. The pellet was gently resuspended in 500 μL of homogenization buffer, loaded onto the surface of a percoll gradient, centrifuged at 1500 g, for 5 min. The green band containing chloroplasts at the interface between the two percoll concentrations was recovered, diluted 4-fold in wash buffer, pelleted at 1000 g for 5 min, resuspended in 500 μL of wash buffer and visualized.

### Microscopy

Leaf tissue and entire seedlings were mounted in deionized water on a glass depression slide and placed under a coverslip for observation using a 40X (numerical aperture 0.80) and 63X (N.A. 0.9) water immersion ceramic lenses. A three channel Leica TCS-SP5 confocal laser- scanning unit equipped with 488nm Argon and 543nm Helium-Neon lasers was used for simultaneous imaging of GFP, YFP, RFP, and chlorophyll at 1 AU pinhole. The emission spectra acquired were: GFP—503 to 515nm (green); RFP- 555 to 630 nm (red); chlorophyll—660 to 750 nm (false colored blue). Images for estimating the size of BnCLIP1-eGFP punctae were acquired using shallow z-stacks of 5 µm with 0.5 µm step-size, acquired at 1 AU pinhole setting. The stacks were collapsed to 2-D images using the maximum projection algorithm of the TCS-SP5 CLSM. Alternatively, single snapshots of 1024 x 512 pixels were taken using 3-line scans at 400Hz and 1 AU.

Observations on transgenic plants expressing tpFNR-EYFP involved independent visualization under a 514 nm line from Argon laser to excite YFP and collecting the 555-600 nm emission spectrum. Simultaneous visualization of GFP, YFP, RFP and chlorophyll was achieved using 488nm and 543nm lines of Argon and Helium-Neon 1, lasers, respectively, and collecting emission spectra between 503-524 for GFP, 555-630 for RFP and 660-750 for chlorophyll. The YFP fluorescence could also be obtained by creating an overlap between the GFP emission and an extended 525-630nm RFP emission spectrum. Although GFP and YFP could be readily distinguished in merged images (**Supplementary Figure S1**) additional confirmation was employed by using the RGB colour sampler tool in Adobe Photoshop CS6 on the acquired images. An equal 250R-250G signifies yellow while 0R-250G signifies pure green, with RGB colour codes of FFFF00 and 00FF00 respectively.

### Photo-conversion of mEosFP

As described earlier (Mathur et al., 2010; Schattat et al., 2012) a five second exposure to a violet-blue glass filter (Leica D-filter; Ex, BP 355–425; dichroic 455; long pass [LP] 470 nm) was used for photo-conversion of the green form of monomeric (m) EosFP (Wiedenmann et al., 2004) to the red form (emission collected 570-620 nm). Since epifluorescence setups usually do not provide beam diameters smaller than 500 µm, smaller pinholes of 100 and 50 µm were custom created on a Leica DMRE epifluorescence microscope (Schattat et al., 2012) for illuminating small regions of interest.

### Imaging parameters

The x,y,z series used for creating 3-D projections maintained a 0.99µm distance between sections. Time-lapse (x,y,t) image capture used a 1024 x 512-pixel box size and a line averaging of three in the bi-directional mode to obtain a frame every 1.935 seconds.

All images were captured at a color depth of 24-bit RGB, cropped and processed for brightness/contrast as complete montages or image stacks using either Adobe Photoshop CS3 (http://www.adobe.com) or the ImageJ/Fiji platform (http://fiji.sc/Fiji). Adobe Photoshop was used for annotation of images and movies.

### Imaging data processing

Kymographs: Using Fiji version 1.53t (https://fiji.sc) a region of interest was defined within the image stack (e.g. rectangular box in **Figure 2H**) to encompass the intended trajectory for generating a kymograph. The built-in Reslice function was applied to the ROI, resulting in a two-dimensional representation that captured the temporal changes along the specified path. The channels were then split to facilitate the observation and analysis of spatiotemporal dynamics pertaining to the investigated phenomena over a period of time.

Pearson’s and Manders’ coefficients were calculated using the JACoP plugin in ImageJ software (https://imagej.nih.gov/ij/). An image sequence comprising every 10^th^ frame of an x,y,t acquisition was analyzed accounting for a total time period of 1 minute and 15 seconds. The colocalization analysis was conducted on images acquired under two distinct channels, with color thresholds set at 90 for BnCLIP1-eGFP (Green channel) and 87 for mRFP-ER (Red channel) across all frames.

For assessing relative areas for chloroplasts and BnCLIP1-eGFP labelled regions (e.g. **Figure 1B**) Z-stacks were acquired via CLSM imaging and subsequently deconvoluted using DeconvolutionLab2 (Version 2.1.2) plugin (Sage et al., 2017) in Fiji, thus enhancing the precision of puncta measurements. The areas of chloroplasts and BnCLIP1-eGFP were quantified by applying the color threshold function in Fiji after channel separation. Twenty-seven chloroplasts each for both epidermal and sub- epidermal chloroplasts, were measured, along with their respective BnCLIP1-eGFP puncta areas. For building up correlations on size (total area) of chloroplasts and their average velocities time-lapse movies of chloroplasts were selected, and velocities were measured using the Manual Tracking plugin in Fiji. Velocity measurements were conducted for 10 individual chloroplasts in each category, encompassing both epidermal and mesophyll plastids. The velocities were averaged for each chloroplast, and these average velocities were plotted against their respective areas in a scatter plot to assess correlation. Error bars, representing the standard deviation in velocity for individual chloroplasts, were also included.

## Data availability statement

The original contributions presented in the study are included in the article/**Supplementary Material**. Further inquiries can be directed to the corresponding author.

## Author contributions

JM: Conceptualization, Funding acquisition, Supervision, Visualization, Writing – original draft, Writing – review & editing. TK: Formal Analysis, Resources, Software, Visualization, Writing – original draft. AM: Investigation, Resources, Validation, Visualization, Writing – original draft. NM: Methodology, Resources, Writing – review & editing.

## Supplementary material

Supplementary Movie 1 Ninety x,y,t frames acquired over 180 seconds played at 5 frames per second show chloroplast envelope localized green-fluorescent patches of BnCLIP1-eGFP. Some patches move away from the main chlorophyll-containing plastid body (false coloured blue).

Supplementary Movie 2 Time lapse series of 55 frames acquired during 110 seconds played at 5 frames per second shows dynamic stochastic localization of green fluorescent BnCLIP-eGFP enriched regions (green) on tubular stromules highlighted (red) using stroma targeted and photoconverted tpFNR-mEosFP. Chlorophyll is false coloured blue.

Supplementary Movie 3 Time-lapse series of 30 frames acquired over 60 seconds, played at 5 frames per second shows face-on view of dynamic red fluorescent ER tubules interacting with envelope localized green fluorescent BnCLIP1-eGFP patch against chlorophyll-containing plastid body (blue). A region of interest (ROI) indicated by the white box subjected to colocalization analysis using the JACoP plugin in Fiji version 1.53t software. Costes automatic threshold values were directly utilized for Pearson’s coefficient analysis. An averaged overall coefficient of 0.93 was obtained.

Supplementary Movie 4 Time-lapse series comprising of 95 frames played at 5 frames per second suggests that interactivity between single, polarly located BnCLIP1-eGFP enriched (green) regions of interaction with the dynamic ER dictates the movement of small chloroplasts (chlorophyll -false coloured blue) in hypocotyl epidermis cells. Note that despite continuous interactivity with the ER not all plastids move at the same time and in the same manner. Accordingly, some plastids might remain confined to a small region of the cell while others move considerable distances while maintaining connectivity with the ER. The differences in plastid movement may be due to regional differences in ER dynamics resulting from ER interaction with cytoskeletal elements.

Supplementary Movie 5 Twenty-three frames spanning 230 seconds showing that ER association and disassociation with BnCLIP1-eGFP highlighted MCS at the envelope are involved in stromule extension and retraction, respectively. A break in connectivity (around frame 7) can be observed leading to the quick retraction of the tubule towards the plastid body.

Supplementary Movie 6 A x,y,t series acquired over 300 seconds shows the rapid shape changes of a chloroplast (chlorophyll false coloured blue) with a tpFNRG-EYFP highlighted stromule. The presence of a BnCLIP1-eGFP (bright green – top panel) puncta creates a small point of interaction with contiguous ER and is implicated in the various contortions. A portion of the stromule beyond the BnCLIP1-eGFP puncta appears to be relatively less affected by the neighbouring ER.
