# Supplementary material for: Membrane contacts with the endoplasmic reticulum modulate plastid morphology and behaviour
Source: Front Plant Sci. 2023 Dec 4;14:1293906. doi: 10.3389/fpls.2023.1293906 (PMC10726010; doi:10.3389/fpls.2023.1293906)

**Supplementary Figure 1: Illustrating independent visualization of YFP, followed by simultaneous visualization of GFP, YFP, RFP and chlorophyll.**

A. Observations on transgenic plants expressing tpFNR-EYFP involved independent visualization under a 514 nm line from Argon laser to excite YFP and collecting the 555-600 nm emission spectrum.

B.C. Simultaneous visualization of GFP, YFP, RFP and chlorophyll was achieved using 488nm and 543nm lines of Argon and Helium-Neon 1, lasers, respectively, and collecting emission spectra between 503-524 for GFP, 555-630 for RFP and 660-750 for chlorophyll. Note distinct GFP fluorescence of BnCLIP1-eGFP. The YFP fluorescence could be obtained by an overlap between the GFP channel and an extended 525-630nm RFP emission spectrum.

D. Simultaneous visualization of YFP-targeted to stroma, green BnCLIP1-eGFP punctae (arrowheads), red fluorescent ER lumen and chlorophyll false coloured blue.

Size bars: A, B, D =5; C=10 µm

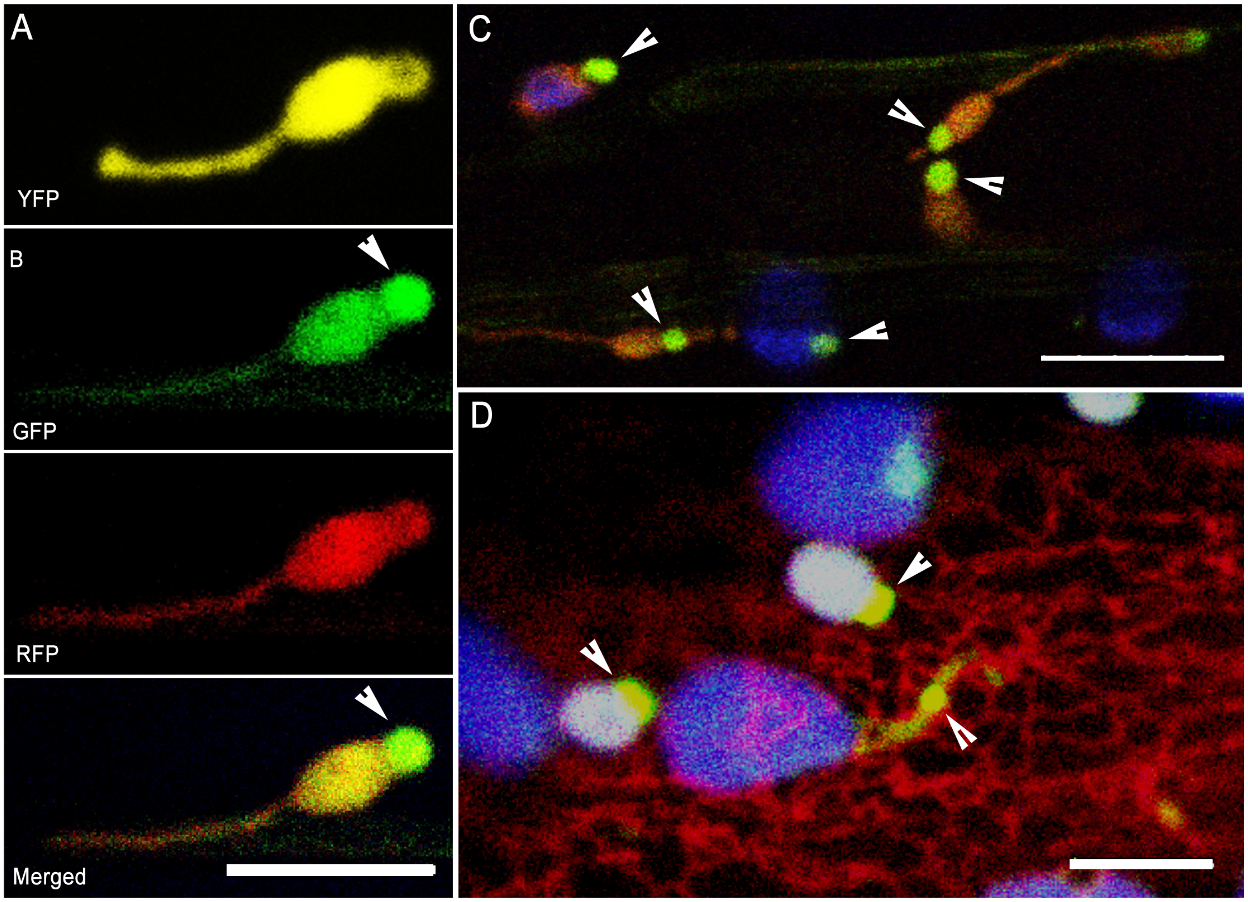

Supplement: Supplementary file 7 [file DataSheet_1.pdf]
